# Block Copolymers of Poly(ω-Pentadecalactone) in Segmented Polyurethanes: Novel Biodegradable Shape Memory Polyurethanes

**DOI:** 10.3390/polym12091928

**Published:** 2020-08-26

**Authors:** Katalin Czifrák, Csilla Lakatos, Marcell Árpád Kordován, Lajos Nagy, Lajos Daróczi, Miklós Zsuga, Sándor Kéki

**Affiliations:** 1 Department of Applied Chemistry, University of Debrecen, Egyetem tér 1, H-4032 Debrecen, Hungary; czifrak.katalin@science.unideb.hu (K.C.); lakatoscsilla@science.unideb.hu (C.L.); kordovan.marci@science.unideb.hu (M.Á.K.); nagy.lajos@science.unideb.hu (L.N.); zsuga.miklos@science.unideb.hu (M.Z.); 2 Department of Solid State Physics, University of Debrecen, Bem tér 18/b, H-4026 Debrecen, Hungary; daroczi.lajos@science.unideb.hu

**Keywords:** copolymerization, ω-pentadecalactone, poly(ω-pentadecalactone), polyurethane copolymers, shape memory

## Abstract

In this report, the synthesis of poly(ω-pentadecalactone) (PPDL) (co)polymers and their incorporation into polyurethanes (PUs) are reported. Optimal conditions for the ring-opening polymerization (ROP) of ω-pentadecalactone (PDL) using dibutyltin dilaurate catalyst were established. For the synthesis of linear and crosslinked PUs, 50 kDa poly(ε-caprolactone) (PCL) and 1,6-hexamethylenediisocyanate (HDI) were used. The obtained polyurethanes were characterized by Attenuated Total Reflectance Fourier-Transform Infrared spectroscopy (AT-FTIR), differential scanning calorimetry (DSC), and dynamical mechanical analysis (DMA). The DMA of the selected sample showed a rubbery plateau on the storage modulus versus temperature curve predicting shape memory behavior. Indeed, good shape memory performances were obtained with shape fixity (R_f_) and shape recovery (R_r_) ratios.

## 1. Introduction

The poly(ω-pentadecalactone) (PPDL) is a novel class of biodegradable polyester polyols. Their biodegradable monomer ω-pentadecalactone (called also as angelica lactone; cyclopentadecanolid; pentadecalactone; pentadecanolide; pentalide; thibetolide) is used as a fragrant ingredient in many cosmetics [1,2]. Similarly to poly(ε-caprolactone) (PCL), the semi-crystalline poly(ω-pentadecalactone) (PPDL) (mp: around 100 °C depending on the molecular weight, T_g_: below −27 °C) also shows large similarity to the crystallization behavior of polyethylene [3,4]. Accordingly, PPDL can be degraded only at stronger hydrolytic conditions (in soil) [5].

The ring opening polymerizations (ROP) catalyzed by enzymes, metal ions, or organic compounds are commonly used for the polymerization of e.g., lactones of various ring-size or other cyclic monomers. However, there is only a limited number of catalysts that are effective for the ROP of large lactones, because the ring strain is not significant, but the entropy plays a more decisive role in the polymerization of these monomers [5,6,7,8,9,10,11].

Based on the literature, it seems that enzyme catalysis is one of the most effective processes leading to high molecular weight poly(ω-pentadecalactone) [3,4,12]. From these, the use of lipase enzyme should be mentioned as active catalyst in PDL polymerization [4,13]. Some metal-based catalysts such as aluminum-salt complexes are also frequently used. They have been shown to be capable of yielding high molecular weight PPDL with M_n_ of 150,000 g/mol [10]. *N*-heterocyclic olefins with simple Lewis acid (e.g., MgCl_2_, LiCl) co-catalyst effectively catalyzed the homo- and copolymerization reactions of ω-pentadecalactone, where the polymerization rate was significantly influenced by metal halide [14]. The strong Lewis acid Al(C_6_F_5_)_3_—in combination with strong Lewis base *N*-heterocyclic olefin—can also facilitate the ring opening (co)polymerization of lactones [15].

The phase-separated structures made up from variable hard and soft segments give a wide variety of properties to the polyurethanes. When the reactants are combined in appropriate ratios, they have beneficial effects on the properties of the resulting polymer, where the hard segments are responsible for the physical crosslinks (Van der Waals interactions and H-bridge) and they can also counteract with the soft segments. The lower temperature properties of the segmented polyurethanes are influenced by the soft segment [16,17,18].

The role of PPDL in the segmented polyurethanes cannot be simply determined since the PPDL segment in these systems can act also as a soft [16] and hard segment [19]. The segmented structure may fulfill the requirement for the construction of t shape memory polymers (SMPs). The SMPs contains net points (hard domains) and switching (e.g., crystalline or semi-crystalline soft segments with a characteristic melting temperature T_m_) elements [20,21]. Introduction of a second crystalline building block into the polyurethane structure, in addition to the mechanical properties, may also influence the shape memory properties as well [16,22]. Thus, it is worth paying more attention to the application of PPDL in the segmented polyurethanes.

This report focuses partly on the optimization of the synthesis of PPDL. For this purpose, in this work, we applied dibutyltin dilaurate as catalyst and diols as initiators in ROP reaction of PDL to obtain PPDL copolymers. On the other hand, this work also aim at the synthesis and characterization of various PPDL containing linear and crosslinked PUs to obtain novel biocompatible and biodegradable shape memory polymers [23]. Therefore, for the initiation of the ROP polymerization of PDL 1,4-butanediol, polyethylene glycol (M_n_ = 200 g/mol) and polycaprolactonediol were applied, which contribute to the flexibility of the resulting polymers [24,25].

## 2. Materials and Characterization

### 2.1. Materials

For poly(ω-pentadecalactone) synthesis, ω-pentadecalactone M = 240.38 g/mol (98%) from Sigma-Aldrich (Darmstadt, Germany) was used as the monomer. As diols and soft segments, 1,4-butanediol (BDO) Reagent Plus^®^ (99%) M = 90.12 g/mol, polyethylene glycol PEG 200 M_n_ = 0.2 kg/mol, poly(ε-caprolactonediol) (PCD) M_n_ = 2 kg/mol, poly(ε-caprolactone) (PCL) from Sigma-Aldrich (Darmstadt, Germany) and CAPA^®^ 6500, M_n_ = 50 kg/mol from Perstop UK Ltd. (Warrington, UK) were used without further purification. Catalysts dibutyltin dilaurate (M = 631.56 g/mol, 95%) and tin(II) 2-ethylhexanoate (M = 405.12 g/mol, 92.5–100%)were used as received from Sigma-Aldrich (Darmstadt, Germany). The 1,6-hexamethylene diisocyanate (HDI) (reagent grades, 98%), tetrahydrofuran (THF) and toluene (reagent grade, 99.7%, distilled over P_2_O_5_ and stored on sodium wire) were purchased from Sigma-Aldrich (Darmstadt, Germany).

### 2.2. Characterization

Size-exclusion chromatography (SEC) was applied to determine the number- (M_n_) and the weight-average molecular weight (*M*_w_) and the polydispersity (*M*_w_/M_n_) of the linear PUs prepared. SEC chromatograms were obtained in tetrahydrofuran (THF) at a flow rate of 0.5 mL/min using a Waters chromatograph equipped with four gel columns (4.6 × 300 mm, 5 µm Styragel columns: HR 0.5, 1, 2 and 4), Waters Alliance 2695 HPLC pump, and with a Waters 2414 refractive index detector (Waters Corporation, Milford, MA, USA). SEC was calibrated using polystyrene standards. The sample was dissolved in THF at a concentration of 5 mg/mL and filtrated by PTFE syringe filter (porosity: 0.45 μm) before injection.

^1^H- and ^13^C-NMR spectra were recorded with a Bruker AM360 (360/90 MHz for ^1^H/^13^C) spectrometer (Bruker Daltoniks, Bremen, Germany). Deuterated chloroform was used as the solvent. Chemical shifts were referenced to the ^1^H signal of Me_4_Si that was used as standard.

The Matrix-Assisted Laser Desorption/Ionization Time-of-Flight Mass Spectrometry (MALDI-TOF MS) measurements were performed using a Bruker Autoflex Speed mass spectrometer equipped with a time-of-flight/time-of-flight (TOF/TOF) mass analyzer (Bruker Daltoniks, Bremen, Germany). For measurements in the positive ion mode, 19 kV acceleration voltage was employed. To get appropriate resolution and mass accuracy, ions were detected in the reflectron mode and 21 kV and 9.55 kV were applied as reflector voltage 1 and voltage 2, respectively. A solid phase laser (355 nm, ≥100 μJ/pulse) working at 500 Hz was used to achieve laser desorption and 5000 shots were summed. The MALDI-TOF MS spectra were externally calibrated with polyethylene glycol standard (M_n_ = 1540 g/mol). Samples for MALDI-TOF MS were prepared with 2,5-dihydroxy benzoic acid (DHB) matrix dissolved in THF at a concentration of 20 mg/mL. The samples and sodium trifluoroacetate used as ionizing agent were also dissolved in THF at a concentration of 10 mg/mL and 5 mg/mL, respectively. The mixing ratio was 10/2/1 (matrix/sample/cationizing agent). A volume of 0.25 μL of the solution was put onto a metal sample plate and left to air-dry.

Attenuated total reflectance (ATR) Fourier-transform infrared (AT-FTIR) spectra were obtained on a Perkin Elmer Instruments Spectrum Two FTIR spectrometer equipped with a diamond Universal ATR Sampling Accessory (PerkinElmer, Waltham, MA, USA). The average film thickness of the specimens was ca. 0.3 mm. Four scans were carried out for each sample.

To get deeper insight into the surface morphology, Scanning Electron Microscopic (SEM) was applied. SEM pictures were taken from the surface of selected specimens by a Hitachi S-4800 microscope (Hitachi, Tokyo, Japan) equipped with a Bruker energy dispersive X-ray spectrometer (Bruker Daltoniks, Bremen, Germany). For microscopic examinations, 1 × 1 cm samples with an average thickness of 0.5 mm were excised. Surface of the specimens was covered with a 30 nm conductive gold layer. The SEM observations were performed at 15 kV accelerating voltage in secondary electron mode.

Tensile tests were performed according to the EN ISO 527-1 standard. Computer-controlled Instron 4302 type tensile testing machine, equipped with a 1 kN load cell, was used. At least three dumbbell specimens were cut (clamped length 60 mm) based on ASTM D882-12 standard and tensile loaded at a crosshead speed of 50 mm/min. The thickness of the specimens varied between 0.3–1.0 mm.

Stress relaxation experiments were performed using an Instron 3366 (Instron, Norwood, MA, USA) type mechanical testing machine. The specimens were elongated to 100% and the decrease of the load at this strain was recorded and evaluated by the Instron Bluehill Universal V 4.05 (2017) software.

The thermal properties of the prepared PUs were investigated by Differential Scanning Calorimetry (DSC). DSC tests were performed in DSC Q2000 power compensation equipment operating at 10 °C/min heating rate (TA Instruments, New Castle, DE, USA). Nitrogen flushing was used as protective atmosphere. During DSC measurements, Heat/Cool/Heat cycles were carried out. Heating cycle: the temperature was raised from −70 °C to 220 °C by 10 °C/min heating rate. Cooling cycle: the sample cooled down from 220 °C to −70 °C using the same heating rate. The weight percentage of the crystalline PCL (C_r_) was calculated by Equation (1) [26]:

(1) $$C_{r}\left(\%\right)=\frac{{\Delta H}_{m}}{\chi_{\text{PCL or PDL}}\cdot {\Delta H}_{m}^{0}}\cdot 100$$

where ΔH_m_ is the heat of fusion of the investigated PU, χ_A_ is the weight fraction of PCL or PDL in the corresponding PU, ΔH_m_^0^ is the heat of fusion of the pure 100% crystalline PCL or PDL [27,28].

Dynamic Mechanical Analysis (DMA) of the PUs was performed by means of a DMA Q800 device (TA Instruments, New Castle, DE, USA). DMA curves were recorded in tension mode (dimension of the specimens: length: 25 mm, clamped length: 12 mm, width: 7 mm, thickness: ca. 0.5 mm) at an oscillation amplitude of 0.2% setting a frequency of 1 Hz and a static load of 1 N. The temperature was varied between 0 °C and 150 °C with a heating rate of 3 °C/min.

Shape memory properties were investigated in tensile mode using the above DMA device. The specimens (clamped length × width × thickness = ca. 12 × 7 × 0.5 mm) were stretched after 10 min holding at 60 °C at a strain rate of 30%/min, followed by quickly cooling the specimen to 20 °C. The stress was then released and the shape fixity (R_f_) determined. Shape recovery (R_r_) was determined at 0.1 N loading of the specimens (quasi free recovery) by reheating the specimens at 1 °C/min heating rate from 20 °C to 60 °C and holding there for 10 min. The shape fixity (R_f_) and shape recovery ratios (R_r_) are given by Equations (2) and (3):

(2) $$R_{f}\left(\%\right)=\frac{l_{d}-l_{o}}{l_{60\%}-l_{o}}\cdot 100$$

(3) $$R_{r}\left(\%\right)=\frac{l_{d}-l_{f}}{l_{d}-l_{o}}\cdot 100$$

where l_d_ is the sample length after removal of the tensile load during shape fixing at 20 °C, l_o_ is the clamped length of the sample at 20 °C, l_60%_ the length after tensile stretching for 60%, and l_f_ the final recovered length of the stretched specimen.

The shape fixation experiments were performed at 20 °C, since this temperature is well below the switching temperatures measured by DSC for PU 4 (65.9 °C for PCL part and 85.5 °C for PPDL part). Due to the flexibility of the 50 kDa PCL segment, 60% tensile stretch was selected for the shape memory measurements.

## 3. Polymerization of ω-Pentadecalactone (PDL) by ROP

**A typical procedure for synthesis of PPDL-1:** The polymerization was carried out in a dried 50 mL round bottom flask equipped with CaCl_2_ tube. To ω-pentadecalactone, 10 g (41.6 mmol) 1,4-butanediol 0.37 g, 0.37 mL (4.16 mmol) dibutyltin dilaurate 0.525 g, 0.50 mL (0.83 mmol, and 2 mol%) were added. The PDL/BDO molar ratio: 10/1. The mixture was kept at 100 °C for 2 days. After cooling to room temperature, the mixture solidified and gave PPDL-1 10.06 g (97%), as pale yellowish powder. The obtained oligo ester was analyzed by ^1^H-NMR and MALDI-TOF MS.

^1^H-NMR characteristics of the monomer and the polymer (product)

PDL monomer: ^1^H-NMR (CDCl_3_, 360 MHz) σ ppm: 4.13 (t, –CH_2_–O–), 2.33 (t, –CH_2_–CO–O–), 1.67–1.59 (m, –C*H_2_*–(CH_2_)_10_–C*H_2_*–), 1.39–1.29 (m, –(CH_2_)_10_–). ^13^C-NMR (CDCl_3_, 90 MHz,) σ ppm: 173.9 (CO), 63.8 (–CH_2_–O–), 34.3, 29.5, 29.2, 28.3, 27.6, 27.0, 26.5, 26.2, 25.9, 25.8, 25.7, 25.0, 24.8 (13 CH_2_).

PPDL-1: ^1^H-NMR (CDCl_3_, 360 MHz) σ ppm: 4.05 (t, –CH_2_–O– PPDL), 3.64 (t, –CH_2_–OH PPDL end group), 2.29 (t, –CH_2_–CO–O–), 1.61–1.58 (m, –C*H_2_*–(CH_2_)_10_–C*H_2_*–), 1.29–1.24 (m, –(CH_2_)_10_–), 0.90–0.87 (m, –(CH_2_)_10_– end group). ^13^C–NMR (CDCl_3_, 90 MHz,) σ ppm: 174.0 (CO), 64.4 (–CH_2_–O–), 34.4 (–CH_2_–CO–O–), 29.61 (2), 29.58, 29.56, 29.5, 29.4, 29.2, 29.1, 28.6, 25.9, 25.0, 24.8 (13 CH_2_).

The number average molecular weight M_n_ and the conversion were determined by ^1^H-NMR spectroscopy as outlined below.

The conversion is given by Equation (4) [29]:

(4) $$\mathrm{Conversion}\;\left(\%\right)=\frac{I_{-{\mathrm{CH}}_{2}O-\mathrm{PPDL}}}{I_{-{\mathrm{CH}}_{2}O-\mathrm{PPDL}}+I_{-{\mathrm{CH}}_{2}\mathrm{OH}-\mathrm{PDL}}}\cdot 100$$

The number average molecular weights were determined by Equations (5) and (6) were used [29]:

(5) $${\mathrm{DP}}_{\mathrm{PPDL}}=\left(\frac{I_{-{\mathrm{CH}}_{2}O-\mathrm{PPDL}}/2}{I_{-{\mathrm{CH}}_{2}\mathrm{OH}-\mathrm{PDL}}/4}\right)+2$$

(6) $$M_{n}=\left({\mathrm{DP}}_{\mathrm{PPDL}}\times M_{\mathrm{PDL}}\right)+M_{\mathrm{end}-\mathrm{group}}$$

where I_-CH2O-_ and I_-CH2OH_ are the signal intensities of the repeat unit and the end-group of PPDL at 4.05 ppm and at 3.64 ppm, respectively. These signals were used to calculate the degree of polymerization (DP). M_PDL_ is the molecular weight of repeating unit of PPDL and M_end-group_ the molecular weight of the initiator moiety (e.g., BDO).

**PPDL-2:** To 10 g (41.6 mmol) ω-pentadecalactone, 0.83 g (4.16 mmol) PEG200 and 0.79 g, 0.75 mL (1.25 mmol, 3 mol%) dibutyltin dilaurate catalyst were added. The reaction mixture was heated at 100 °C for 2 days. The applied PDL/PEG 200 molar ratio was 10/1. 10.59 g (98%) white powder formed after cooling. ^1^H-NMR (CDCl_3_, 360 MHz) σ ppm: 4.05 (t, –CH_2_–O– PPDL), 3.70–3.63 (t, –CH_2_–OH PPDL end group and –CH_2_– of PEG), 2.29 (t, –CH_2_–CO–O– PPDL), 1.61–1.58 (m, –C*H_2_*–(CH_2_)_10_–C*H_2_*– PPDL), 1.29–1.24 (m, –(CH_2_)_10_– PPDL), 0.92–0.88 (m, –(CH_2_)_10_– PPDL in end group). ^13^C–NMR (CDCl_3_, 90 MHz,) σ ppm: 174.0 (CO, PPDL), 63.4 (–CH_2_–O–, PPDL), 34.4 (–CH_2_–CO–O–, PPDL), 29.62 (3), 29.6 (3), 29.52, 29.46, 29.3 (2), 29.2, 28.6, 25.9, 25.0 (14 CH_2_, PPDL and PEG).

**PPDL-3:** Prepared from 3.0 g (1.5 mmol) poly(ε-caprolactonediol, M_n_ = 2000 g/mol) (PCD), 3.61 g (15 mmol) ω-pentadecalactone and 0.284 g, 0.270 mL (0.45 mmol, 3 mol%) dibutyltin dilaurate catalyst. The polymerization reaction was kept at 100 °C for 2 days. The applied PDL/PCD molar ratio was 10/1 to give 6.55 g (99%) amorphous white material. ^1^H-NMR (CDCl_3_, 360 MHz) σ ppm: 4.07–4.04 (m, –CH_2_–O–PPDL and –CH_2_–O– PCD), 3.64 (t, –CH_2_–OH PPDL end group), 2.32–2.26 (m, –CH_2_–CO–O– PPDL and PCD), 1.64–1.59 (m, –C*H_2_*–(CH_2_)_10_–C*H_2_*– PPDL and –C*H_2_*–CH_2_–C*H_2_*– PCD), 1.39–1.37 (m, –CH_2_– PCD), 1.27–1.24 (m, –(CH_2_)_10_– PPDL), 0.93–0.88 (m, –C*H_2_*–CH_2_–C*H_2_*– PCD and –(CH_2_)_10_–, PPDL end group). ^13^C–NMR (CDCl_3_, 90 MHz,) σ ppm: 174.0, 173.9 (CO PCD, PPDL), 64.5, 64.4, 64.1, 64.0 (–CH_2_–O–, PCD, PPDL), 34.4, 34.3, 34.14, 34.10 (–CH_2_–CO–O–, PCD, PPDL), 29.61(2), 29.57 (2), 29.51, 29.45, 29.2 (2), 29.1, 28.6, 28.3, 25.9, 25.5, 24.99, 24.96, 24.7, 24.55 (CH_2_).

## 4. Synthesis of PUs

**A typical procedure for synthesis of PU 1:** In a previously dried three-necked flask equipped with condenser and a CaCl_2_ guard tube, 3 g (0.06 mmol) PCL (M_n_ = 50 kg/mol) was dissolved in 30 mL dry hot toluene and 30 μL (0.18 mmol, Equation (3)) 1,6-hexamethylene diisocyanate (HDI) was added in the presence of 3 drop tin(II) 2-ethylhexanoate catalyst. The reaction mixture was stirred at 100–110 °C for 2 h. To the reaction, 0.3 g (0.06 mmol, Equation (1)) PPDL-1 with M_n_ = 4890 was added in one portion. After further stirring for one night at 80 °C, the mixture was poured into Teflon plate and dried at room temperature to give an elastic polymer film.

PUs 2 and 3 were synthesized according to the same procedure.

The crosslinking reactions for the synthesis of PUs 4–6 were carried out by adding additional one equivalent HDI (10 μL, 0.06 mmol) to the reaction mixture and stirred further for 1 h at the same temperature. Then the mixture was poured into a Teflon plate and dried at room temperature, resulting in the formation of a polymer film.

The compositions of samples PU 1–PU 6 are compiled in Table 1.

## 5. Results and Discussion

### 5.1. Synthesis and Characterization of α, ω-PPDL Diols by ROP

**Scheme 1:**
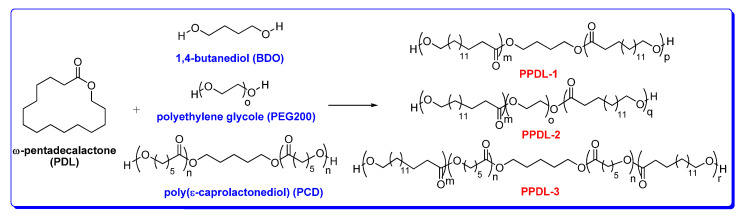
Reaction scheme for the synthesis of PPDL 1–3.

To polymerize PDL to obtain PPDL with high conversion, dibutyltin dilaurate was used as catalyst and 1,4-butanediol (BDO), polyethylene glycol (PEG 200) and poly(ε-caprolactonediol) (PCD) were used as initiators (Scheme 1).

During the ring opening polymerization (ROP), the dibutyltin dilaurate catalyst facilitates the opening of the macro lactone ring by activating the carbon atom of the carbonyl group [12,30].

**Figure 1:**
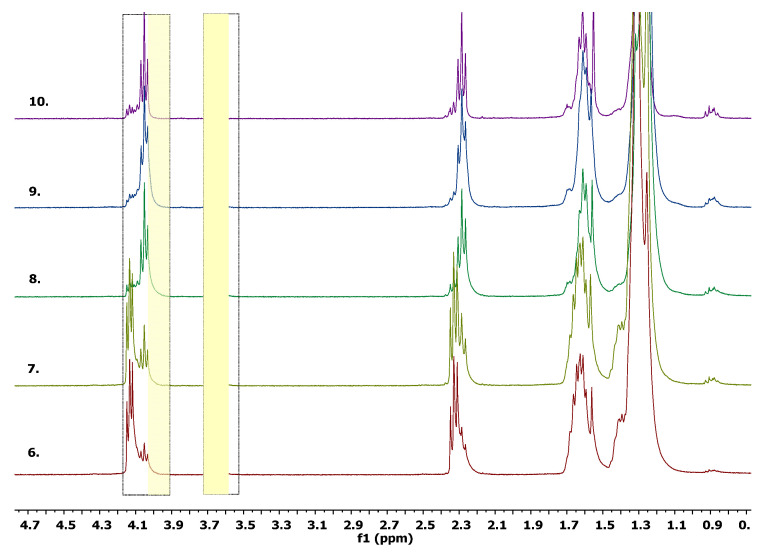
Superimposed ^1^H-NMR spectra of the products formed in the reactions 6–10 (see Table 2) recorded in CDCl_3_.

The reaction conditions were optimized in the presence of the BDO initiator at 100 °C in bulk polymerizations (Table 1) and monitored by ^1^H-NMR (Figure 1). Based on the results, the PDL/BDO ratio does not significantly affect the conversion, which ranged from 9 to 13% (Table 1, rows 1–5).

Therefore, in further studies, the PDL/BDO ratio was selected as 10/1 and the amount of catalyst was increased (Table 2, rows 6–10). The highest conversion, degree of polymerization, and molecular weight were found in the presence of 2 mol% dibutyltin dilaurate.

It should also be noted that tin(II) 2-ethylhexanoate, which is more reactive than dibutyltin dilaurate in the polymerization of PDL, yielded PPDL-s as a white insoluble powder with high molecular weight.

Based on the results of ROP of PDL with BDO short chain diol as an initiator, the optimized reaction conditions were also employed for PEG200 and PCD initiators (Table 3). The corresponding ^1^H-NMR spectra are shown in the Supporting Information as Figure S1. In the latter polymerization reactions, 3 mol% catalyst was used to obtain high conversion.

**Figure 2:**
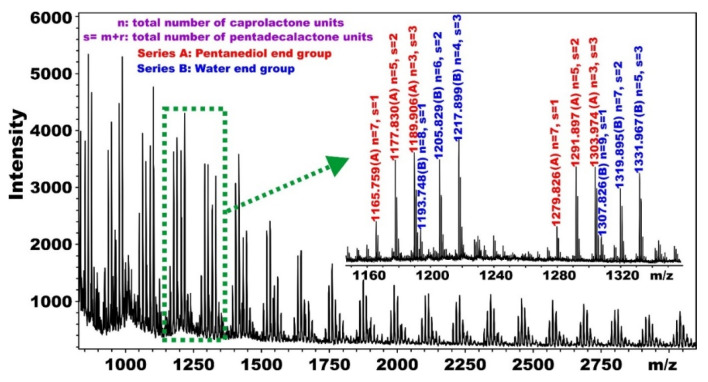
MALDI-TOF MS spectrum of sample PPDL-3. The numbers in the brackets represent the numbers of the caprolactone and pentadecalactone repeat units. The isotopic distribution, measured and calculated (green in brackets) *m*/*z* values are shown in the inset (top right corner).

In order to confirm that successful initiation from the targeted initiators took place, MALDI-TOF MS measurements were performed (Figure 2).

In the range of *m*/*z* 1000–2750, the MALDI-TOF MS spectrum shows the presence of two main series. As illustrated in the top right corner of Figure 2, series A belongs to the PPDL-PCD-PPDL block copolymer in which the number of caprolactone units varies between 3–7, while the pentadecalactone number changes between 1 and 3. Series B represents the PPDL-PCL-PPDL block copolymer, the formation of which can be explained by the fragmentation of PCD. In the cases of PPDL-1 and PPDL-2, in addition to the target copolymers, homopolymers of PDL can also be observed with low intensity (see Figures S2 and S3 in Supporting Information).

### 5.2. Synthesis and Characterization of PPDL Containing Urethanes

**Scheme 2:**
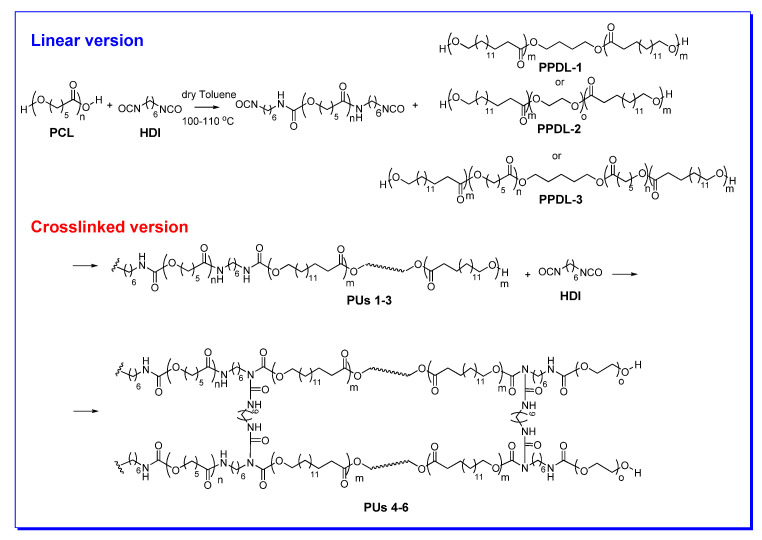
Reaction schemes for the synthesis of PUs 1–6.

During the synthesis of PUs 1–3, PCL with 50 kg/mol molecular weight was reacted first with HDI in ratio 1/3 [31] followed by addition of PPDLs, as depicted in Scheme 2. PUs 2, 3 and 5, 6 contain PPDL-2 and PPDL-3 unit, respectively, in which the longer chain diol initiator can offset the stiffening effect of PPDL (Scheme 1 and Table 2). The crosslinking between the linear polymer chains was achieved by adding another equivalent diisocyanate (HDI) to the reaction mixture (see PUs 4–6).

### 5.3. Infrared Spectroscopy (FT-IR)

**Figure 3:**
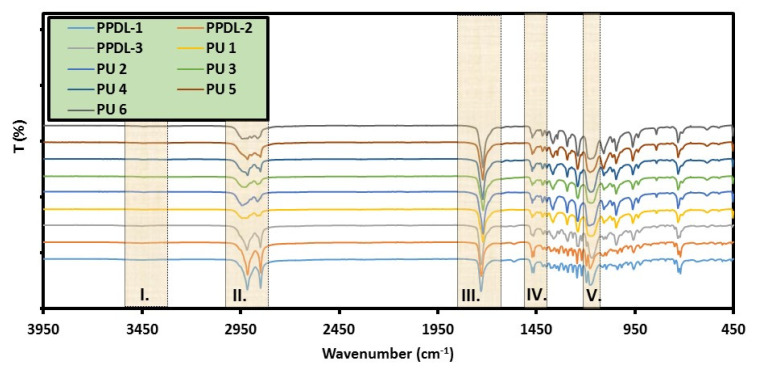
ATR-FTIR spectra of the synthesized PPDL and PU samples in the range of 450–3950 cm^−1^. The shaded areas represent the characteristic bands.

Further information on the chemical structure can be obtained from the IR spectra recorded on the PPDL copolymers and polyurethanes PUs 1–6 (Figure 3). The low intensity absorption band around 3440 cm^−1^ (**I.**) belongs to the amide and/or urethane –NH bands. The signal of –CH_2_ vibrations in the IR spectra of PPDL-1–3 copolymers appears as a double sharp band at 2915 and 2849 cm^−1^ (**II.**) indicating the presence of a large number of CH_2_ groups in these polymers. However, in samples PU 1–6, this band is more complex and occurs between 2942–2849 cm^−1^. The lack of the absorption band around 2230 cm^−1^ refers to the complete reaction of the NCO functional groups. The H-bonded structure characteristic of urethanes can also be detected by IR-spectroscopy. The free C=O band reflecting the ester (PCL/PPDL unit) and urethane C=O appears around 1723–1720 cm^−1^, whereas the H-bonded C=O band occurs as a weak shoulder between 1714–1685 cm^−1^ (**III.**). However, for PPDL 1–3, the C=O band can be found at 1730 cm^−1^. The amide II band at 1515 cm^−1^ is not detectable, but the band at around 1470 cm^−1^ (**IV.**) appears in all spectra. The peak at around 1180 cm^−1^ (**V.**) can be assigned to the =C–O and –C–O–C– bonds. The zoomed parts of Figure 3 are presented in Supporting Information (see Figures S4–S6).

### 5.4. Morphology of PUs by SEM

**Figure 4:**
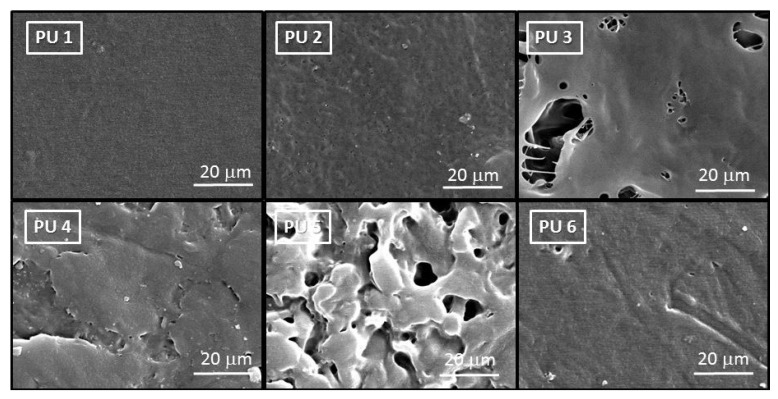
SEM pictures of samples PU 1–6 for visualization of the morphology.

The SEM images of PU samples are shown in Figure 4.

As seen in Figure 4, similar, but not identical microstructures were formed. Furthermore, there is no deep inclusion and no significant phase separation occurred in samples PUs 1, 2 and 6. These findings may confirm that the polymers have a regular structure. On the contrary, the morphologies of samples PUs 3–5 do not seem to be uniform, which may be due to fact that crosslinking causes microphase separation (see e.g., PUs 4 and 5).

### 5.5. Mechanical Properties of PUs

In order to obtain a deeper insight into the mechanical properties of the PU samples, uniaxial tensile measurements and stress relaxation experiments were performed. The results of the tensile measurements for the samples PU 1–6 are summarized in Table 4. The tensile diagrams for PUs 1–6 are presented in Supporting Information (see Figures S7–S12).

As turns out from Figures S7–S12, the stress-strain curves are unusually complicated, indicating that the physical state of the samples is very complex. The PU-s prepared contain high molecular weight (M_n_ = 50 kg/mol) polycaprolactone (PCL) in addition to the PPDL segment; therefore, the crystallinity of the samples may change during the tensile test. This is supported by the whitening of the samples during the tests. Thus, the presence of long PCL segments not only increases the elasticity (and thus elongation), but also result in a series of hardening stages during the test.

In the light of the tensile test data of the linear PUs 1–3 containing PPDL, the polypentadecalactone unit seems to be the hard segment in addition to the urethane block, which stiffens the polymer structure. This is partly supported by the variation of the mechanical properties when diol initiators with longer chain are used in the PPDL segment, which might work as a plasticizer. As a result, the long-chain initiator moieties act as soft segments, the E-modulus decreased from 427 to 292 MPa, while the ultimate elongation and stress at break (σ_R_) increased from 428% to 749% and from 21.5 to 31 MPa, respectively. Note that PU 4, PU 5, and PU 6 are the crosslinked versions of PU 1, PU 2, and PU 3, respectively (see Table 1).

Crosslinking with HDI influences the mechanical properties, mainly the stress and the elongation at break values as well as the value of E-modulus. Interestingly, the σ_R_ values do not vary significantly with crosslinking in the case of PU 2–PU 5 and PU 3–PU 6 pairs, while σ_R_ show considerable increase for PU 1–PU 4 pair. This finding might suggest an effective crosslinking in the case of PU 4. In addition, in the cases of PU 1–PU 4 and PU 2 –PU 5 pairs, the E-modulus decrease with crosslinking, which might be attributed to the fact that crosslinking may suppress the crystallization of the PPDL segments to some extent yielding moduli of lower values. A similar finding has been obtained for the linear/crosslinked polyethylene [32,33]. On the contrary, the crosslinked versions have higher ε_R_ values than those of the linear ones. This apparent contradiction can be explained as the result of hindering the movement of the whole polymer chains by crosslinking, and thus, allowing a longer elongation for the sample before it breaks apart.

**Figure 5:**
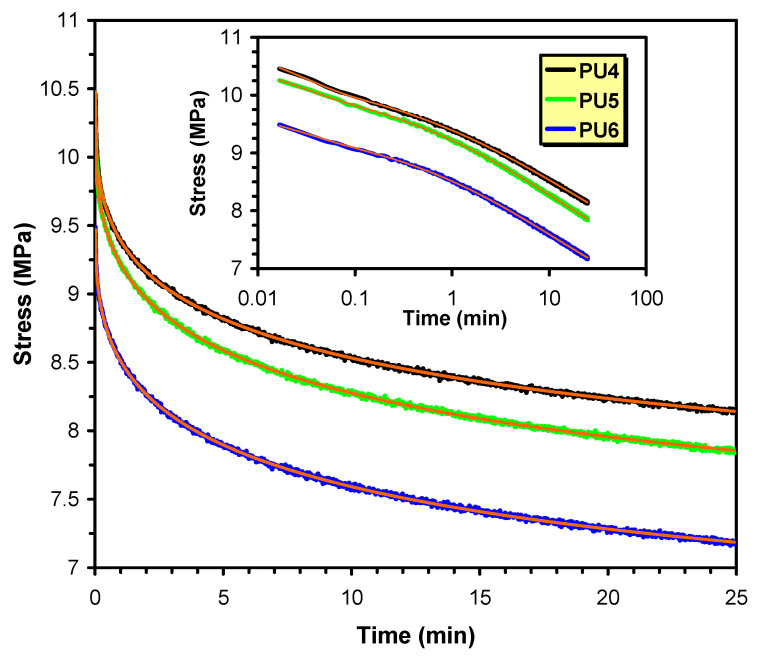
Stress (σ) versus time relaxation curves for the samples PUs 4–6 (initial elongation was 100%). The inset shows the corresponding stress (σ) versus time relaxation curves on a logarithmic *x*-axis scale. The orange solid lines represent the fitted curves by using Equation (7). The fitted parameters for PU 4 are A_1_ = 0.51 MPa, A_2_ = 3.61 MPa, A_3_ = 7.0 MPa, τ_1_ = 0.027 min, τ_2_ = 17.7 min and λ = 0.33, for PU 5 are A_1_ = 0.35 MPa, A_2_ = 3.56 MPa, A_3_ = 6.8 MPa, τ_1_ = 0.027 min, τ_2_ = 13.5 min and λ = 0.35, and for PU 6 are A_1_ = 0.29 MPa, A_2_ = 3.28 MPa, A_3_ = 6.3 MPa, τ_1_ = 0.027 min, τ_2_ = 11.9 min and λ = 0.37.

Stress relaxation experiments were also performed for the crosslinked PU samples (PUs 4–6). The results of the stress relaxation experiments are shown in Figure 5.

As can be seen in Figure 5 and Figure 5 inset, all the stress relaxation curves reveal a fast stress decay at the beginning followed by a slower, stretched relaxation process. Furthermore, it is also evident from Figure 5. that variation of the stress versus time follow the order of PU 4 > PU > 5 > PU 6 in line with the order of crosslink densities.

To describe the stress versus time relaxation curves, a model containing a simple, exponential fast relaxation mode and a stretched exponential decay mode was implemented (Equation (7)):

(7) $$\sigma \left(t\right)=A_{1}\cdot e^{-\frac{t}{\tau_{1}}}+A_{2}\cdot e^{-{\left(\frac{t}{\tau_{2}}\right)}^{\lambda }}+A_{3}$$

where A_1_ and A_2_ are the extent of the fast exponential and the stretched exponential decay, respectively, A_3_ is the final (equilibrium) stress, τ_1_ and τ_2_ being the relaxation time for the fast and the slow decay, respectively and λ represents the “the stretching factor” of the slow decay.

As seen in Figure 5 and Figure 5 inset, very good match were obtained for all the three PUs with similar parameters. However, both the σ (4.12, 3.91 and 3.57 MPa) and A_3_ (7.0, 6.8 and 6.3 MPa) values decreased in the order of PU 4 > PU 5 > PU 6 in good agreement with the experimental ones.

### 5.6. Thermal, Thermomechanical, and Shape Memory Behavior

**Figure 6:**
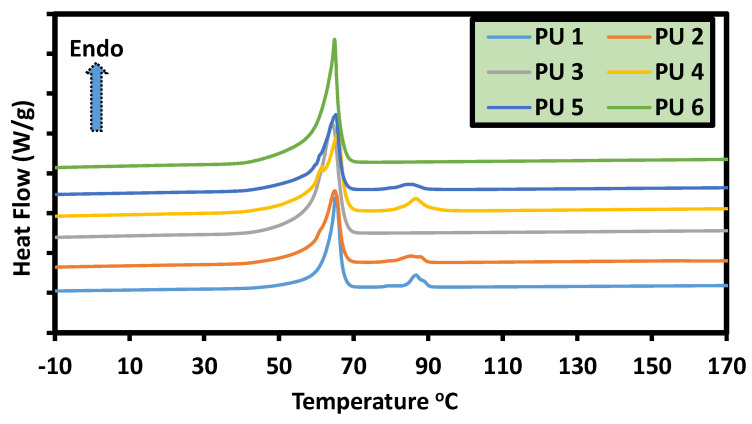
First heating DSC curves of PUs 1–6 presented in the temperature range from −10 °C to 170 °C.

The thermal behaviors of PUs were investigated by DSC. The reversible main “switch on” function of this series of PUs is due to the melting of the PCL segments. On the other hand, the PPDL segment may partly influence the crystallization of the PCL segment and thus, it may affect the shape memory properties. For this reason, the crystallization behavior of PCL in the selected PUs 1–6 was studied by DSC (Figure 6). The crystallinity values were calculated based on the mere PCL and PDL weight content in each copolymer, using as the reference ΔH_m_^0^ value of PCL and PPDL [28]. The crystallinity values were determined separately for the PCL and PPDL segments. The data obtained from the DSC curves and the calculated crystallinity values are summarized in Table 5.

As seen in Figure 6, in addition to the melting peak of PCL at around 65 °C, another small peak at ca. 87 °C, attributed to the presence of PPDL segments can be detected by DSC. Based on the ΔH_m_ values of PCLs shown in Table 5, PPDL affects the crystallinity of PCL and acts as a self-supporting crystalline phase. The exception to that are the PU 3 and PU 6 samples containing the PPDL-3 segment. In these samples, the relatively low molecular weight polycaprolactone segment (M_n_ = 2000 g/mol) may hinder the crystallization of the PPDL block, giving rise to the absence of endothermic peak at ca. 80–90 °C.

On the other hand, crosslinking also affects the crystallinity of both the PCL and the PPDL segments. In the case of pairs of PU 1–PU 4 and PU 2–PU 5, crosslinking decreases the extent of crystallinity in good agreement with the E-modulus values, as discussed earlier (Table 4).

**Figure 7:**
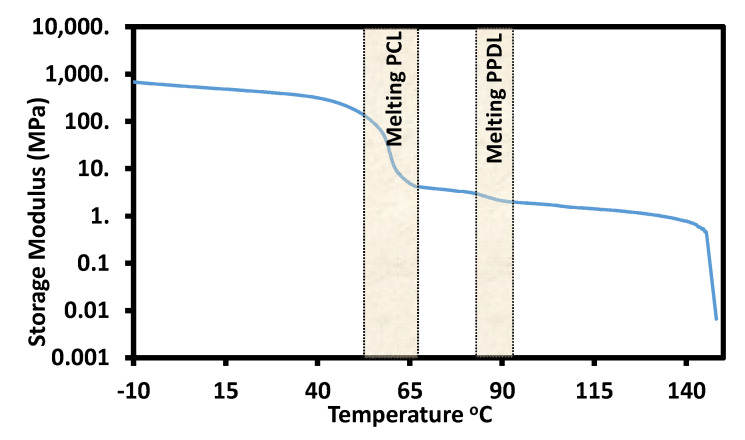
Storage modulus as a function of temperature for PU 4. The shaded areas show the meltings of the PCL and the PPDL segments.

The thermomechanical behaviors of PUs 4–6 were also studied by means of dynamic mechanical analysis (DMA) in tensile mode (Figure 7).

As seen in Figure 7, the storage modulus (E′) versus temperature curve shows a relatively large decrease around 60–65 °C and an another, smaller drop in the storage modulus at ca. 80–90 °C. As we have seen earlier, these two transition temperatures are in line with the melting of the PCL (60–65 °C) and the PPDL (80–90 °C) segments. Furthermore, an unambiguous rubbery plateau appeared, which indicates the presence of a crosslinked structure in PU 4. However, no clear rubbery plateaus could be detected on the DMA curves of PU 5 and PU 6 due to the breakaway of these samples. Using Equation (8), and the storage modulus value at the onset of rubbery state (90 °C), one can also calculate the crosslink density, for which 2.32 × 10^−4^ mol/cm^3^ was obtained:

(8) $$\nu_{e}=\frac{E^{\prime }}{3\mathrm{RT}}$$

where E′, R, and T are the storage modulus, the universal gas-constant, and the temperature at the onset of the rubbery state, respectively.

**Figure 8:**
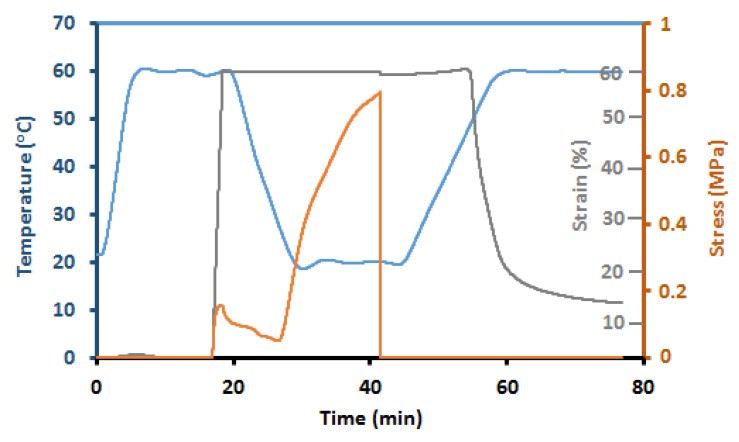
Temperature versus time, strain versus time (shape recovery) and stress versus time curves for PU 4 at 60 °C with an initial strain of 60%.

Based on the results of DMA, it can be concluded that crosslinks will provide a permanent shape, while T_m_ of the PCL segments at around 60 °C will serve as a switching temperature for the transition from the temporary to the permanent shape. Accordingly, the shape memory effect for PU 4 was tested in uniaxial tensile mode (Figure 8).

Indeed, as shown by Figure 8, the shape memory test demonstrates relatively good shape fixity (R_f_) and shape recovery (R_r_) ratios, which were determined to be 99% and 81%, respectively.

In addition, an attempt was also made to describe the shape recovery process using a two-exponential decay function considering a quasi isotherm recovery process (Equation (9)):

(9) $$\varepsilon \left(t\right)=A_{f}\cdot e^{-\frac{t-t_{o}}{\tau_{f}}}+A_{s}\cdot e^{-\frac{t-t_{o}}{\tau_{s}}}\;(t\geq t_{o})$$

where A_f_ and A_s_ are the extents of the exponential decay with relaxation times τ_f_ and τ_s_.

**Figure 9:**
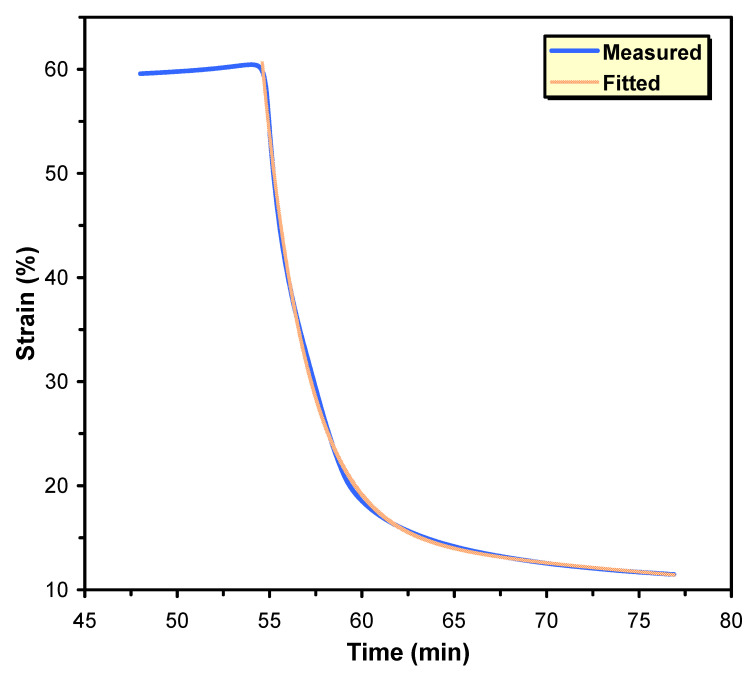
Variation of the strain with time for the shape recovery of PU 4. Initial strain: 60%. The reddish solid line shows the fitted curve using Equation (9). The fitted parameters are A_f_ = 41.4%, A_s_ = 15.3%, τ_f_ = 2.4 min and τ_s_ = 76.3 min. Fitting was started at t_0_ = 54.6 min.

As Figure 9 demonstrates, the curve calculated by Equation (9) match well with the experimental one.

The fast relaxation term of the two-exponential function (τ_f_ = 2.4 min) describes the fast relaxation process associated with the melting of the PCL segment (enthalpy driven process), while the larger relaxation time (τ_s_ = 76.3 min) may indicate a slow relaxation process for recovering the original conformations of the polymer chains (entropy driven process). The two-exponential function has been successfully applied for the description of shape recovery for other PU systems [34].

## 6. Conclusions

The polymerization of ω-pentadecalactone (PDL) by ROP was optimized and monitored by means of ^1^H-NMR spectroscopy and MALDI-TOF MS spectrometry. Applying appropriate reaction conditions, three PPDL (co)polymers were synthesized and incorporated into linear and crosslinked polyurethanes. For the determination of the details of the structure of PPDL, MALDI-TOF MS was applied. The synthesized PUs were investigated by thermal, thermomechanical (DSC, DMA), and mechanical methods in order to get information on the effect of PPDL unit on the properties of these PU polymers. Based on the FTIR spectra of PPDL-containing polyurethanes, the presence of PPDL segments could not be unambiguously demonstrated due to its structural similarity to the PCL. However, the effect of PPDL unit on the properties of the PU samples can be clearly justified by the results of the mechanical and thermomechanical tests. In light of the tensile test results, in the synthesized PUs, the polypentadecalactones act as the hard segment, stiffening the polymer structures. The presence of the PPDL segment in the PU samples was also supported by DSC measurements. According to the results of DMA obtained on PU 4 above T_m_ of the PCL, a clear rubbery plateau occurred, making its shape memory programming possible. Furthermore, incorporation of PPDL unit into the PUs can provide an additional switch in addition to the crystalline PCL segments, carrying a possibility for multi-shape memory programming.

## Figures and Tables

**Table 1:** The compositions of samples PU 1–PU 6.

Code	Composition in the Feed/Molar Ratio
PU 1	PCL(50)-HDI-(PPDL-1)/1-3-1
PU 2	PCL(50)-HDI-(PPDL-2)/1-3-1
PU 3	PCL(50)-HDI-(PPDL-3)/1-3-1
PU 4	PCL(50)-HDI-(PPDL-1)-HDI/1-3-1-1
PU 5	PCL(50)-HDI-(PPDL-2)-HDI/1-3-1-1
PU 6	PCL(50)-HDI-(PPDL-3)-HDI/1-3-1-1

**Table 2:** The applied PDL/initiator ratio, catalyst amount, obtained conversion, polymerization degree, and molecular weight determined by NMR of PDL polymerization.

Sample	PDL/Initiator (mol ratio) ^a^	Catalyst (mol%)	Conv. (%) ^b^	DP ^b,c^	M_n_ (NMR) ^b,c^
1	10/1	0.1	9.1	4.4	1150
2	20/1	0.1	12.2	6.5	1650
3	30/1	0.1	13.5	18.2	4460
4	40/1	0.1	12.9	12	2970
5	50/1	0.1	10.8	17.4	4270
6	10/1	0.5	24	10	2490
7	10/1	1.0	31.4	12.4	3070
8	10/1	1.5	80.6	22	5370
9	10/1	2.0	85	22	5370
10	10/1	2.5	80	20.2	4940

^a^ Polymerizations were carried out at 100 °C for 24 h with 2.5 mmol of PDL; PDL/initiator (BDO) molar ratio were changed in 5 experiments. Dibutyltin dilaurate was used as catalyst. ^b^ Determined by ^1^H-NMR in CDCl_3_. Mn (g/mol). ^c^ Obtained from Equation (5).

**Table 3:** The conversion, DP_n_ and M_n_ values for the samples PPDL-1, PPDL-2, and PPDL-3 determined by ^1^H-NMR and GPC.

Sample	Initiator ^a^	Conv.(%) (NMR) ^b^	DP_n_	M_n_ (NMR) ^c^	M_n_ (GPC)	*M*_w_/M_n_
PPDL-1	BDO	92.6	20	4890 *	-	-
PPDL-2	PEG200	~100	12	2990 *	-	-
PPDL-3	PCD	~100	-	-	5880 **	1.5

^a^ Polymerizations at 100 °C for 48 h with a 10/1 PDL/initiator molar ratio. Dibutyltin dilaurate were used as catalyst. ^b^ Determined by ^1^H-NMR in CDCl_3_. ^c^ Obtained from Equation (6) [29]. * Could not be determined by GPC due to the insolubility in THF. ** Could not be determined by ^1^H-NMR due to the overlapping methylene signals of PEG and PPDL.

**Table 4:** Tensile mechanical properties of the PUs (PU 1–6). Designations: E-elastic modulus (Young’s modulus), σ_m_ maximum stress, ε_m_ elongation at the maximum stress, ε_R_ is the ultimate elongation, and σ_R_ is the stress at break.

Code	E (MPa)	ε_m_ (%)	σ_m_ (MPa)	ε_R_ (%)	σ_R_ (MPa)
PU 1	427 ± 52	8.6 ± 1.8	18.5 ± 5.4	428 ± 26	21.5 ± 3.2
PU 2	397 ± 35	9.9 ± 0.4	18.5 ± 1.9	596 ± 125	29.3 ± 4.1
PU 3	292 ± 4	9.4 ± 0.1	15.3 ± 0.3	749 ± 13	30.8 ± 1.1
PU 4	235 ± 17	11.5 ± 1.3	12.4 ± 0.8	892 ± 122	29.9 ± 3.2
PU 5	329 ± 35	10.6 ± 0.2	15.9 ± 1.3	627 ± 127	26.2 ± 1.5
PU 6	334 ± 2	8.9 ± 0.5	14.8 ± 1.1	756 ± 126	27.2 ± 0.5

**Table 5:** Melting temperature (T_m_), melting enthalpy (ΔH_m_), and crystallinity values obtained from DSC measurements for PCL and PPDL segments in PUs 1–6 by analysis of the first heating cycle.

Code	T_m_(°C)	H_m_ (J/g)	Cr(PCL) (%)	T_m_(°C)	H_m_ (J/g)	Cr (PPDL) (%)
PU 1	65.3	87.7	70.7	86.7	9.7	91.6
PU 2	64.9	84.4	66.7	85.5	6.8	87.8
PU 3	64.2	96.0	76.0	-	-	-
PU 4	65.9	75.5	61.0	86.5	8.7	83.4
PU 5	65.2	84.8	65.6	85.4	5.0	64.5
PU 6	64.9	104.4	82.8	-	-	-

## Supplementary Materials

The following are available online at https://www.mdpi.com/2073-4360/12/9/1928/s1, Figure S1: The proton spectra of PDL (1); PPDL-1 (2); PPDL-2 (3) and PPDL-3 (4). Figure S2: MALDI-TOF MS spectrum of PPDL-1. Figure S3: MALDI-TOF MS spectrum of PPDL-2. Figure S4: Magnified detail I. of IR spectrum PPDL 1–3 copolymers and PUs 1–6. Figure S5: Magnified detail II. of IR spectrum PPDL 1-3 copolymers and PUs 1–6. Figure S6: Magnified detail III. of IR spectrum PPDL 1-3 copolymers and PUs 1–6. Figure S7: Tensile diagram of PU 1. Figure S8: Tensile diagram of PU 2. Figure S9: Tensile diagram of PU 3. Figure S10: Tensile diagram of PU4. Figure S11: Tensile diagram of PU 5. Figure S12: Tensile diagram of PU 6.
