# Supplementary material for: Block Copolymers of Poly(ω-Pentadecalactone) in Segmented Polyurethanes: Novel Biodegradable Shape Memory Polyurethanes
Source: Polymers (Basel). 2020 Aug 26;12(9):1928. doi: 10.3390/polym12091928 (PMC7563291; doi:10.3390/polym12091928)
Supplement: Supplementary file 1 [file polymers-12-01928-s001.pdf]

Block copolymers of poly(ω-pentadecalactone) in segmented polyurethanes: Novel biodegradable shape memory polyurethanes

Katalin Czifrák[1], Csilla Lakatos[1], Marcell Árpád Kordován[1], Lajos Nagy[1], Lajos Daróczi[2], Miklós Zsuga[1], Sándor Kéki[1]

[1] Department of Applied Chemistry, University of Debrecen, Egyetem tér 1, H-4032 Debrecen, Hungary

[2] Department of Solid State Physics, University of Debrecen, Bem tér 18/b, H-4026 Debrecen, Hungary

Table of contents

Figure S1. The proton spectra of PDL (1); PPDL-1 (2); PPDL-2 (3) and PPDL-3 (4)............3

Figure S2. MALDI-TOF MS spectrum of PPDL-1…………………………………………......4

Figure S3. MALDI-TOF MS spectrum of PPDL-2……………………………………...……4

Figure S4. Magnified detail I. of IR spectrum PPDL 1-3 copolymers and PUs 1-6………….5

Figure S5. Magnified detail II. of IR spectrum PPDL 1-3 copolymers and PUs 1-6…………5

Figure S6. Magnified detail III. of IR spectrum PPDL 1-3 copolymers and PUs 1-6……..…6

Figure S7. Tensile diagram of PU 1……………………………………………………..7

Figure S8. Tensile diagram of PU 2……………………………………………………..7

Figure S9. Tensile diagram of PU 3……………………………………………………..8

Figure S10. Tensile diagram of PU 4…………………………………………………....8

Figure S11. Tensile diagram of PU 5……………………………………………………9

Figure S12. Tensile diagram of PU 6……………………………………………………9

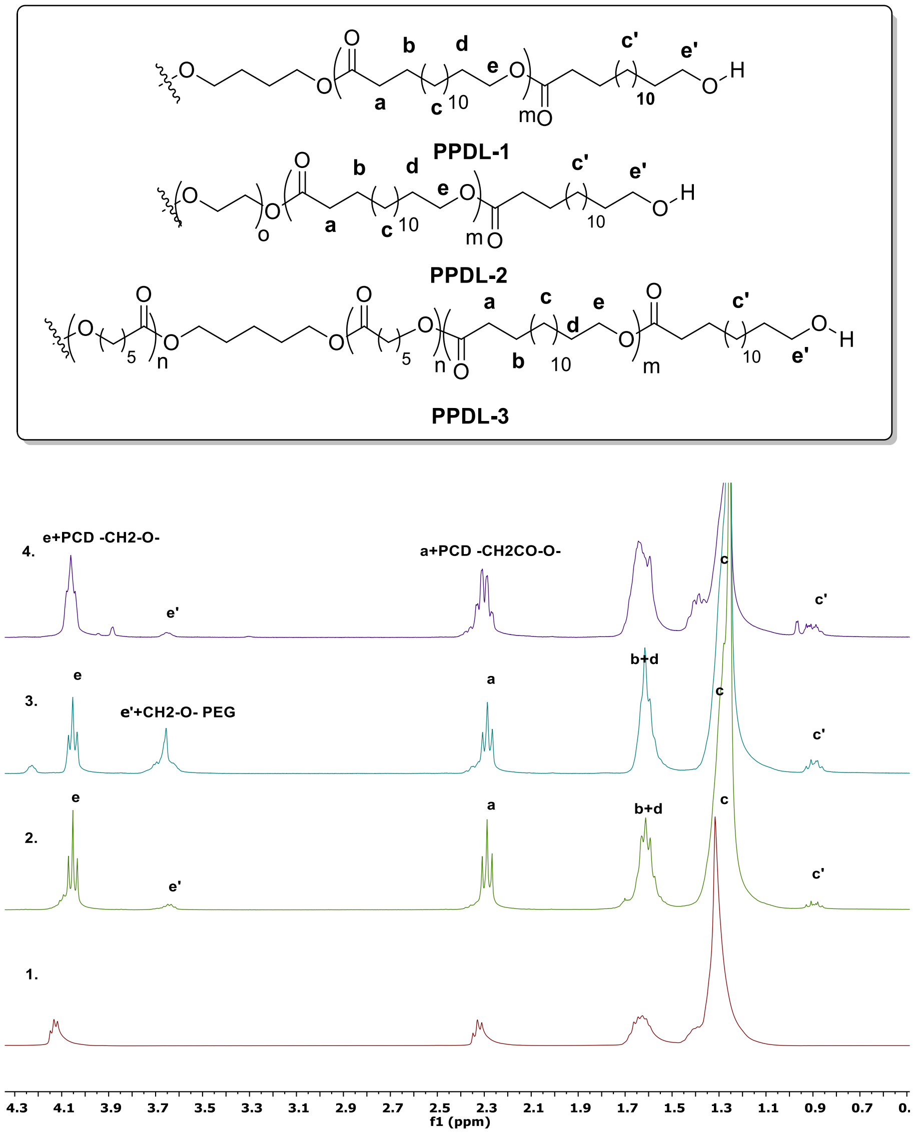

Figure S1. The proton spectra of PDL (1); PPDL-1 (2); PPDL-2 (3) and PPDL-3 (4).

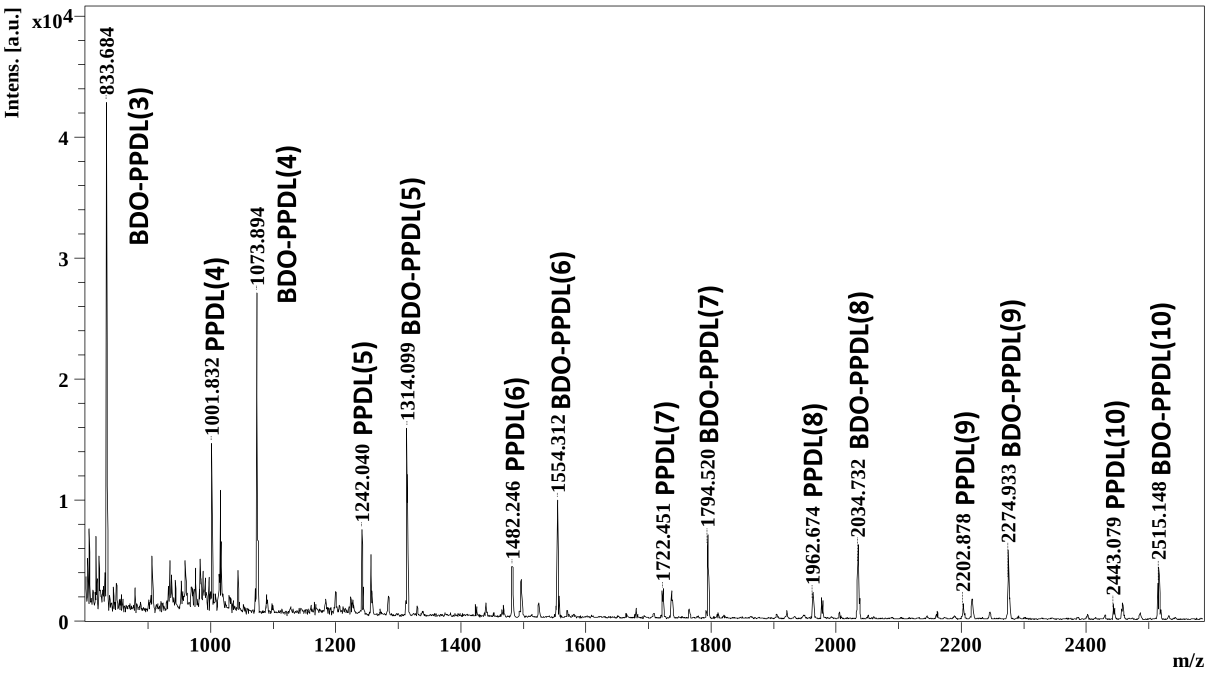

Figure S2. MALDI-TOF MS spectrum of PPDL-1.

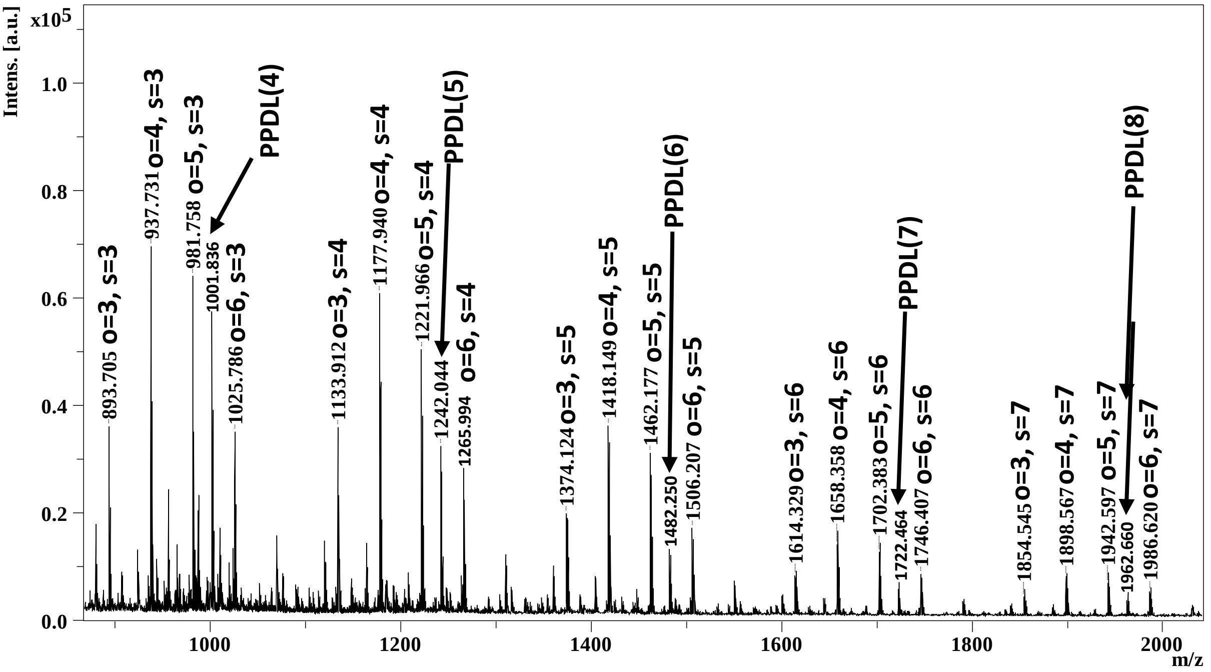

Figure S3. MALDI-TOF MS spectrum of PPDL-2.

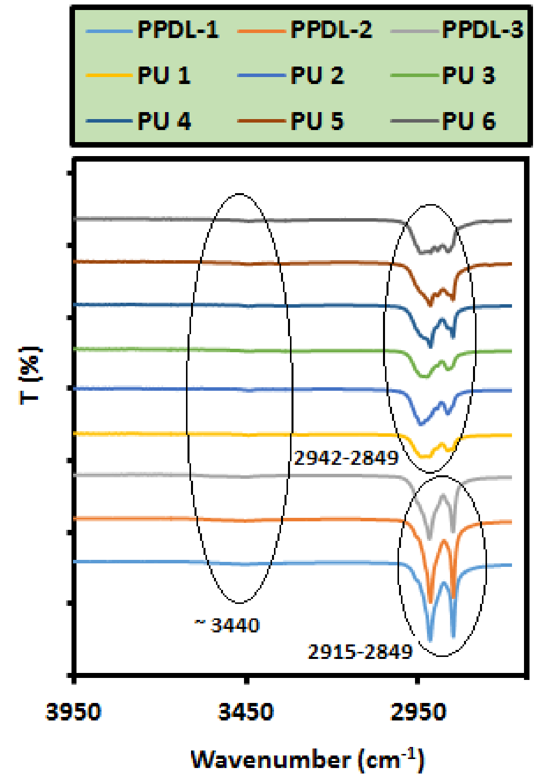

Figure S4. Magnified detail I. of IR spectrum PPDL 1-3 copolymers and PUs 1-6

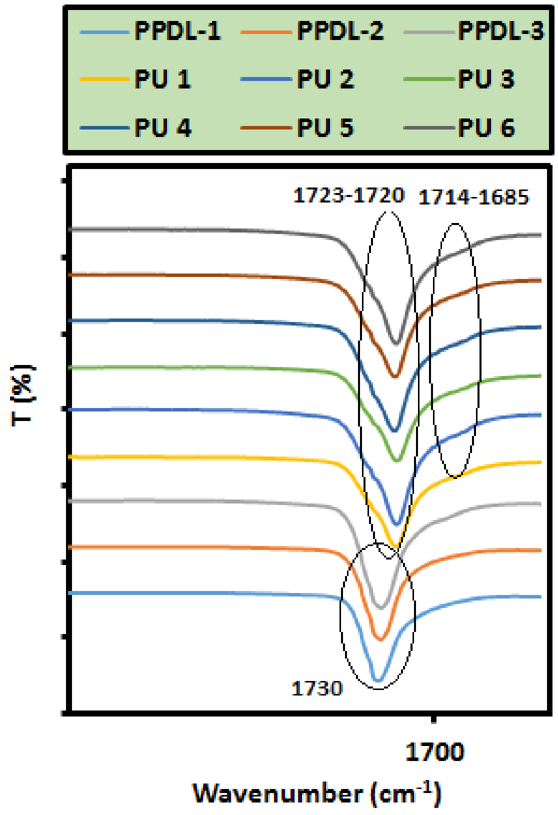

Figure S5. Magnified detail II. of IR spectrum PPDL 1-3 copolymers and PUs 1-6

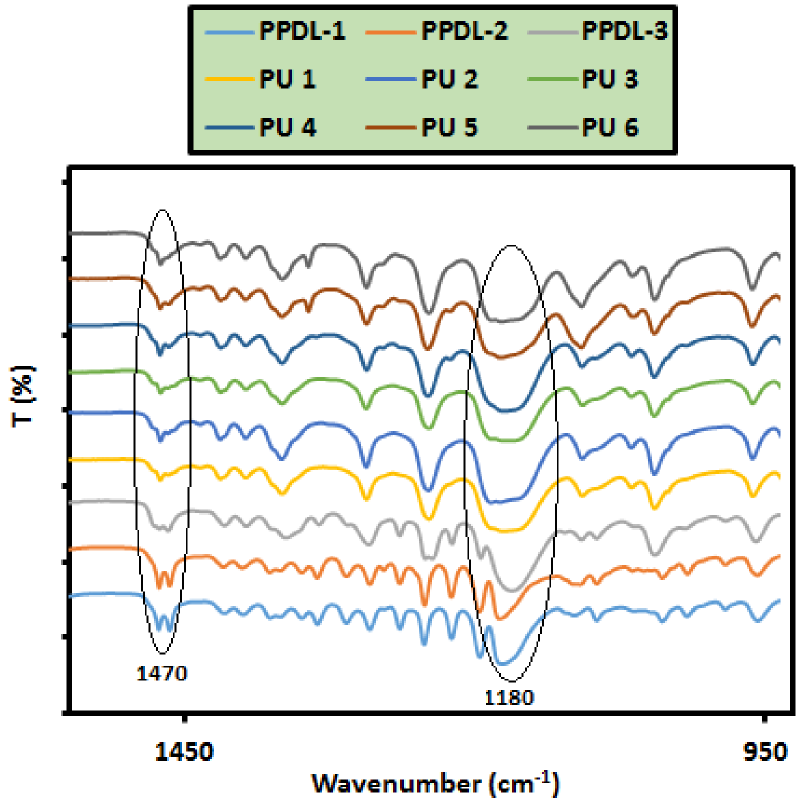

Figure S6. Magnified detail III. of IR spectrum PPDL 1-3 copolymers and PUs 1-6

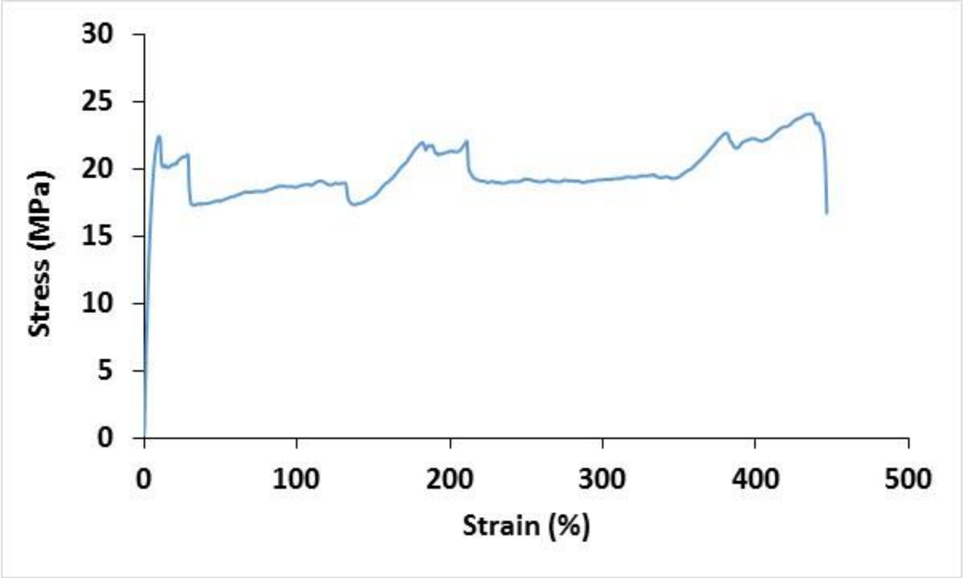

Figure S7. Tensile diagram of PU 1

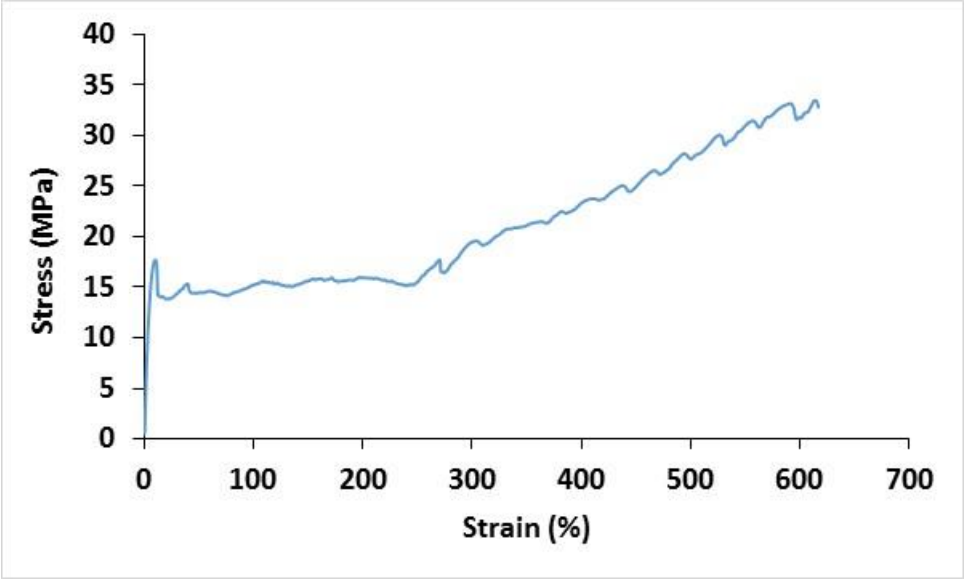

Figure S8. Tensile diagram of PU 2

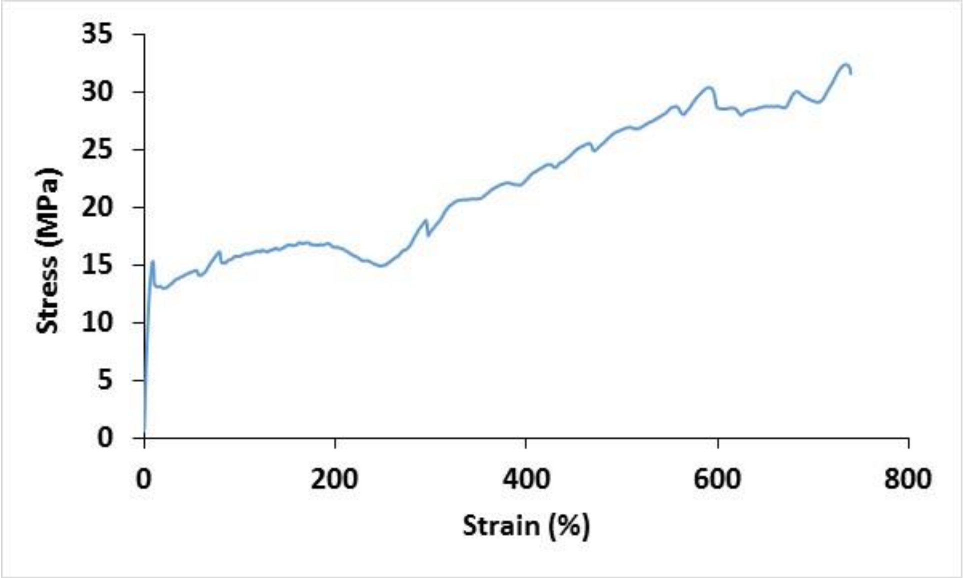

Figure S9. Tensile diagram of PU 3

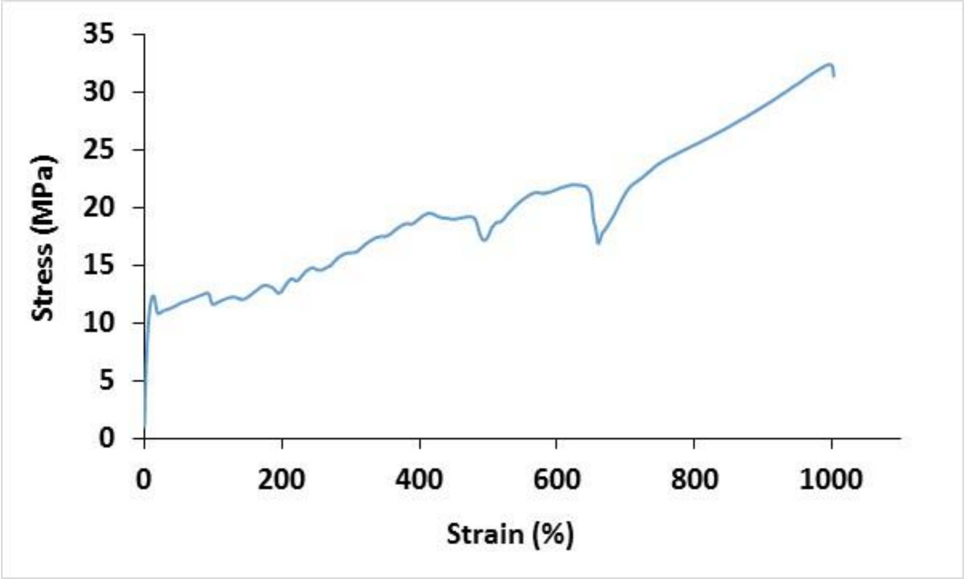

Figure S10. Tensile diagram of PU 4

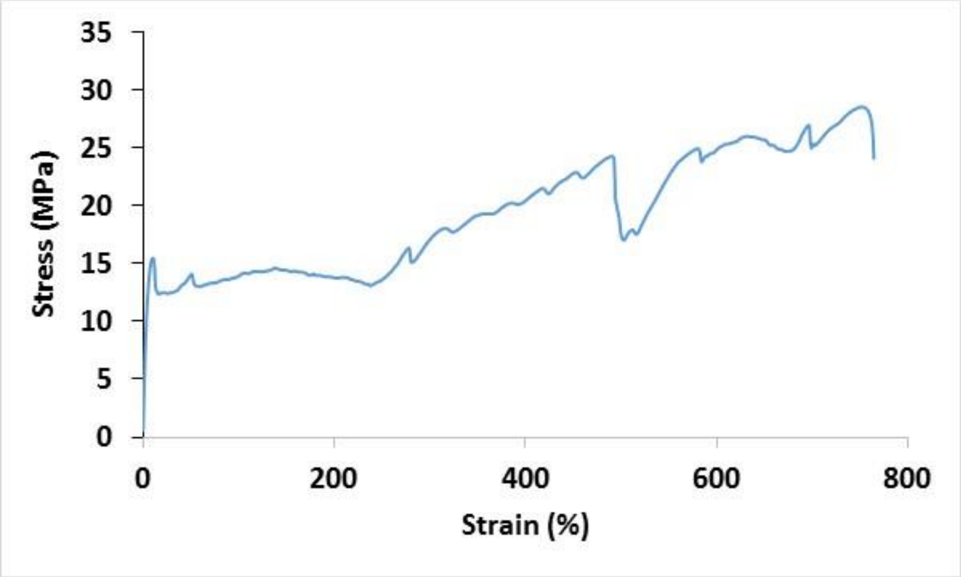

Figure S11. Tensile diagram of PU 5

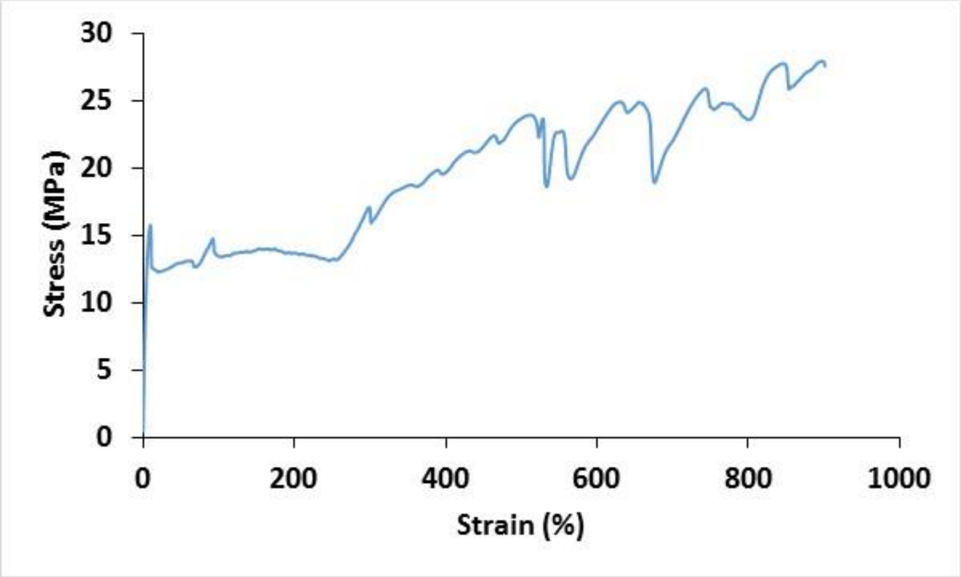

Figure S12. Tensile diagram of PU 6
